# Computational counterselection identifies nonspecific therapeutic biologic candidates

**DOI:** 10.1016/j.crmeth.2022.100254

**Published:** 2022-07-11

**Authors:** Sachit Dinesh Saksena, Ge Liu, Christine Banholzer, Geraldine Horny, Stefan Ewert, David K. Gifford

**Affiliations:** 1 Computer Science and Artificial Intelligence Laboratory, Massachusetts Institute of Technology, Cambridge, MA 02139, USA; 2 Computational and Systems Biology, Massachusetts Institute of Technology, Cambridge, MA 02139, USA; 3 Department of Electrical Engineering and Computer Science, Massachusetts Institute of Technology, Cambridge, MA 02139, USA; 4 Department of Biological Engineering, Massachusetts Institute of Technology, Cambridge, MA 02139, USA; 5 Novartis Institute of BioMedical Research (NIBR), Basel, Switzerland

**Keywords:** biologics, antibody discovery, screening, developability, machine learning, counterselection, nonspecificity, polyspecificity, affinity selection

## Abstract

Effective biologics require high specificity and limited off-target binding, but these properties are not guaranteed by current affinity-selection-based discovery methods. Molecular counterselection against off targets is a technique for identifying nonspecific sequences but is experimentally costly and can fail to eliminate a large fraction of nonspecific sequences. Here, we introduce computational counterselection, a framework for removing nonspecific sequences from pools of candidate biologics using machine learning models. We demonstrate the method using sequencing data from single-target affinity selection of antibodies, bypassing combinatorial experiments. We show that computational counterselection outperforms molecular counterselection by performing cross-target selection and individual binding assays to determine the performance of each method at retaining on-target, specific antibodies and identifying and eliminating off-target, nonspecific antibodies. Further, we show that one can identify generally polyspecific antibody sequences using a general model trained on affinity data from unrelated targets with potential affinity for a broad range of sequences.

## Highlights

• Computational counterselection identifies nonspecific antibodies in candidate pool
• ML models of affinity can be trained for use in computational counterselection
• Nonspecificity in antibody libraries can be driven by generally polyspecific sequences
• Computational counterselection can identify generally polyspecific sequences

## Motivation

Biologics, such as monoclonal antibody therapeutics, are routinely discovered via screening large, randomly diversified libraries for promising sequences. While these methods are effective for identifying candidates with high affinity for targets of interest, they require the use of molecular counterselection for identifying nonspecific binding, which utilizes combinations of selected unintended targets and can lack sensitivity. The nonspecific binding of therapeutics can lead to costly failure during drug development and unintended adverse health effects. We sought to develop a computational method for identifying nonspecific antibody candidates early in the process without combinatorial experiments by training machine learning models on single-target sequencing data from antibody affinity-selection campaigns.

## Graphical abstract

**Figure:**
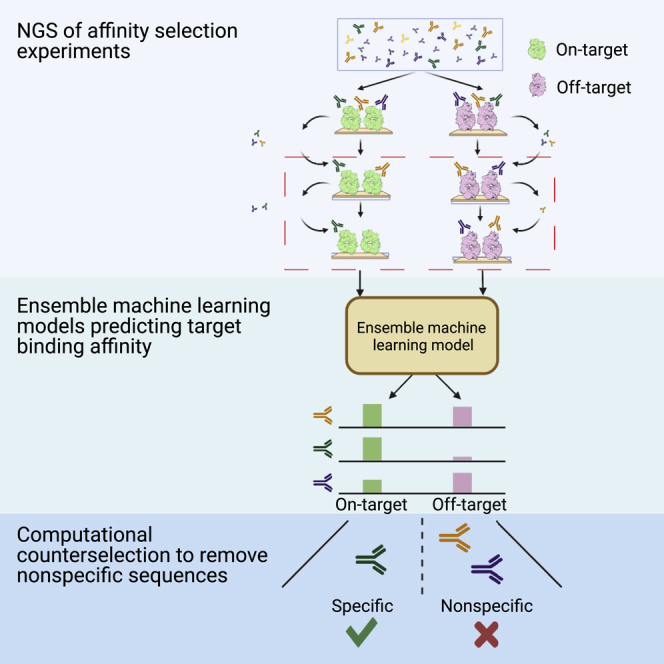

## Teaser

Biologics require high specificity for targets, but current affinity-selection-based discovery methods do not guarantee this property. Saksena et al. present a method, computational counterselection, that identifies nonspecific candidates using machine learning models of affinity trained on high-throughput data from single-target affinity selection experiments.

## Introduction

Biologics have increasingly become an important therapeutic modality in the treatment of cancer, infectious diseases, and other human diseases. A growing number of biologic therapeutics, primarily biological sequences such as proteins or aptamers, are discovered using affinity-selection techniques in which large libraries of candidate sequences are screened, or “panned,” against a desired target, and strong binders are identified as lead candidates for further preclinical development. This technique is useful but often results in a large proportion of unusable candidates due to nonspecific interactions with potential off targets that cannot be evaluated during single-target screens. This often results in significant wasted resources on high-affinity binders that are ultimately undevelopable due to nonspecificity. Here, we present a framework for using high-throughput sequencing from affinity-selection campaigns to computationally identify and filter nonspecific sequences, increasing the efficiency of early-stage therapeutic discovery. We showcase the utility of this approach applied to antibody therapeutic discovery, but it can be used for any sequence-based biologic discovery campaign that uses affinity-based screening.

The high-affinity binding of synthetic antibodies to disease related targets has provided an important source of therapeutics, and the safety of these therapeutics relies in part upon their ability to bind a single desired target and, more importantly, avoid nonspecific binding. In one application, therapeutic antibodies are clinically used to block and activate cellular receptors. In other applications, when conjugated with other bioactive substances, antibodies can implement a wide range of therapeutic modalities (An, 2010). The nonspecific binding of antibodies can result in negative consequences ranging from limited therapeutic efficacy to illness and death (Raybould et al., 2019; Zhou et al., 2007). Thus, antibody specificity is crucial.

Affinity-selection techniques are often used to screen libraries against targets of interest, and molecular competitors can be included to reduce the probability that affinity-selected antibodies will bind to predetermined potential off targets (Chiu et al., 2019). This approach, molecular counterselection, relies upon the accurate selection of the undesired target and its concentration. Thus, molecular counterselection is specific to one or more predetermined off-target molecules, and thus the data from molecular counterselection for a target cannot be used to reduce undesired binding to untested off-target molecules. Further, molecular counterselection is inherently combinatorial—each potential set of off targets requires a separate counterselection experiment, which is in the limit intractable when considering a large number of potential off targets. In practice, candidate antibodies are assayed further down the antibody discovery pipeline for binding to undesired targets using a battery of *in vitro* high-throughput-array-based assays and adverse effects in animal studies. This can result in significant wasted resources on high-affinity antibody candidates that are ultimately found to be unusable. More recently, experimentally designed libraries that attempt to minimize the promiscuous binding of antibodies have been proposed by excluding certain trinucleotide combinations during random synthesis, but this approach relies on a small set of deterministic rules that cannot capture all aspects of nonspecific binding (Kelly et al., 2017). Small-scale computationally designed libraries with individually specified sequences have also been proposed, but achieving the diversity necessary for therapeutic discovery with libraries composed of directly synthesized sequences is expensive and currently intractable (Kosuri and Church, 2014; Liu et al., 2020; Shin et al., 2021).

We introduce computational counterselection, a general method that utilizes sequencing data from affinity-selection experiments to train machine learning models of nonspecific binding. An attractive aspect of this approach is that historical affinity data can be collectively repurposed to improve the detection of off-target binders for future affinity-selection-based discovery campaigns. In the work presented here, we train models using the affinity enrichment of antibody heavy-chain complementarity determining region 3 (CDR-H3) sequences. Using these models, for a given antibody’s CDR-H3 sequence, we predict affinity for the on-target of interest and the set of off-targets. Since sequencing of affinity-selection experiments is now routinely performed, sequencing data from antibody discovery campaigns for a wide range of targets are continuously being generated and can be used to train computational counterselection models. We focus on variation in the CDR-H3 sequence here because this region has been found to exhibit the largest sequence and conformational diversity of the CDRs and has been shown to drive specificity of the antibody binding domain (D’Angelo et al., 2018). We note that computational counterselection could be expanded to include variation in other CDRs with selection data from libraries that have diversified other CDRs. In the absence of antibody campaigns for relevant targets, we demonstrate that using data from affinity-selection experiments against targets commonly used to assay polyspecificty can identify generally nonspecific sequences. Other methods have been proposed that predict specificity using machine learning approaches but only use single-target affinity measures for screening large libraries for specific binding without considering combinatorial specificity against suspected off targets (Mason et al., 2021). Computational counterselection is an explicit tool for nonspecificty identification that can be used in an iterative loop with routinely performed affinity-selection experiments of on- and off targets of interest, providing an avenue for highly certain filtering of nonspecific sequences early in the discovery process.

## Results

### Multi-task neural network ensembles predict binding affinity to trastuzumab and omalizumab

**Figure 1:**
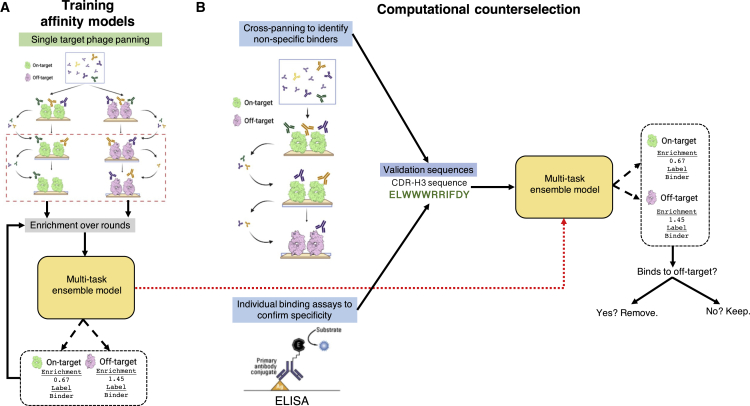
Overview of computational counterselection strategy and experimental validation (A) Using enrichment over rounds from single-target phage panning as a regression label, we train multi-task ensemble models that jointly predict affinity to on and off targets. We can then use this affinity prediction to identify sequences that bind to the off-target molecule and remove these sequences. (B) Comparison with molecular counterselection for validation. To compare to molecular counterselection, cross-panning experiments of the on and off target and individual binding assays of 48 selected sequences were done.

First, we trained models on sequencing data from phage panning against two individual targets, omalizumab (Xolair) and trastuzumab (Herceptin). We chose two publicly available molecules that had both unique and shared epitopes for our evaluation of computational counterselection. We reasoned that the inclusion of shared epitopes would provide a natural source of nonspecific binding, increasing the difficulty of counterselection. We combined the single-target panning data for omalizumab and trastuzumab via a full outer join (i.e., the union of sequences in both datasets) and trained a multi-task ensemble that predicts round 2 (R2) to R3 enrichment of sequences binding to these two targets (Figure 1A). We choose ensemble models, as they provide an explicit measure of epistemic uncertainty, which is essential when training with noisy and potentially sparse experimental sequencing data. Multi-task learning allows for soft parameter sharing between on- and off-target predictions, which improves the ability for the models to learn shared features driving nonspecificity. Because the union of the two datasets includes non-overlapping sequences (i.e., sequences present in one dataset but not the other) between the two datasets, we used a masked mean-squared-error loss to deal with the missing values during training. Masking occurred when updating target-specific weights when data for that target was not observed for a given input sequence in the training set (STAR Methodss; Table S1). When evaluated on held-out validation data, this multi-task ensemble successfully predicts binding affinity of trastuzumab (r = 0.65) and omalizumab (r = 0.59) (Figure S1). Further details on data preprocessing and training datasets are provided in the STAR Methods.

### Machine-learning-guided computational counterselection eliminates antibodies with affinity for both trastuzumab and omalizumab

Next, we utilized these multi-task neural network models to conduct computational counterselection to identify nonspecific sequences that bind both trastuzumab and omalizumab (Figure 1A). Computational counterselection uses multi-task binding-affinity models of on- and off targets and identifies nonspecific sequences if the predicted enrichment for off targets by the model is above a chosen threshold (Figure S2). We then validated this approach using experimental data from both cross-panning experiments and individual binding assays of specificity (Figure 1B). Briefly, cross-panning is a phage selection experiment in which the first two rounds of panning are conducted against the on-target molecule and the last round is conducted against the off-target molecule. Sequences enriched in the third round of cross-panning experiments share affinity to both the on- and off targets, and we classify them as nonspecific antibody sequences.

**Figure 2:**
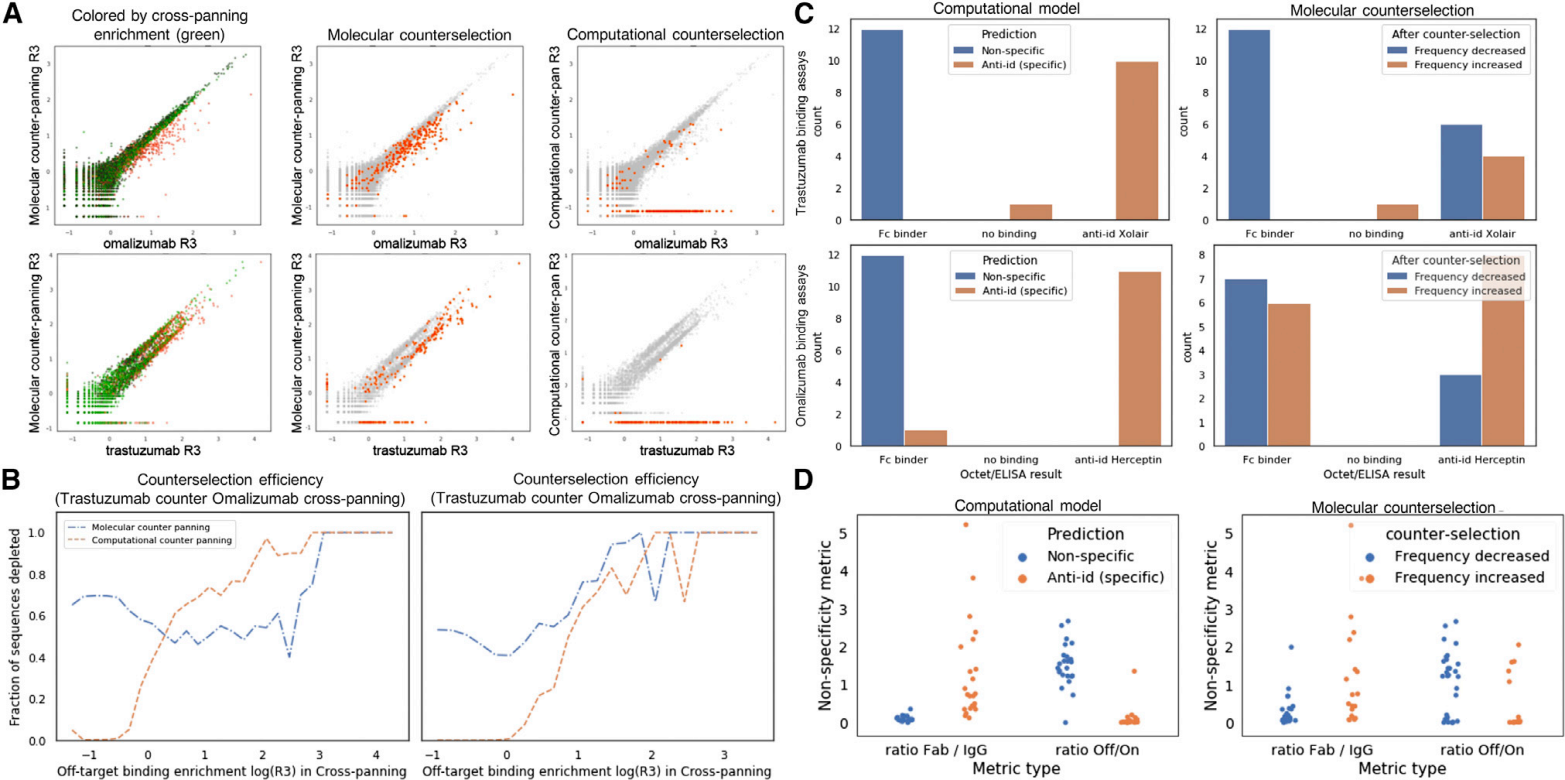
Computational counterselection outperforms molecular counterselection in removing off-target antibodies from antibody libraries (A) Computational counterselection removes off-target binders more effectively than molecular counterselection. The x axis is the antibody enrichment for the on-target antigen. The y axis is the antibody enrichment for the on-target antigen in the presence of an off-target competitor (counterselection). In the leftmost scatterplots, points are colored by their enrichment in independent cross-panning experiments (green). Across all plots, off-target antibody sequences are identified by independent cross-panning and are strictly highlighted in orange. The middle plots show depletion of off-target sequences by molecular counterselection. The far-right plots show off-target sequences identified and set to zero by computational counterselection. (B) Computational counterselection (orange) is more efficient than molecular counterselection across off-target affinity levels, leading to fewer false positives and negatives. The y axis is the efficiency of nonspecific binder removal, and the x axis is the independent observation of enrichment of nonspecific binding in a cross-panning experiment. Computational counterselection (orange) and molecular counterselection (blue) curves are shown. (C) Computational counterselection provides superior classifications using ground-truth metrics from Octet/ELISA data for 48 candidates split by trastuzumab (top) and omalizumab (bottom) predictions. Computational counterselection and molecular counterselection predictions indicated by blue (nonspecific) and orange (anti-idiopathic) bars grouped by ground-truth labels. (D) Fab/immunoglobulin G (IgG; lower ratio indicates nonspecific binding) and cross-selection/on-target selection ratios (higher indicates nonspecific binding). Computational counter-selection (left) and molecular counterselection (right) predictions indicated by blue (nonspecific) and orange (specific) were evaluated by their Fab/IgG and cross-selection/on-target selection ratio distributions.

We found that computational counterselection was more effective at reducing off-target binding than conventional molecular counterselection when cross-panning was conducted with both trastuzumab as the on-target and omalizumab as the off-target, and vice versa. Efficient counterselection should reduce nonspecific binding by a significant margin. We found that molecular counterselection failed to eliminate a substantial fraction of off-target binding. In contrast, we found that computational counterselection succeeded in removing most nonspecific binders (Figure 2A).

We next quantified the efficiency of nonspecific antibody removal as a function of an antibody’s cross-panning R3 frequency. High R3 antibody sequence frequency in a cross-panning experiment indicates that an antibody sequence is nonspecific, as it will only be observed if it binds to both antigens. For molecular counterselection, we consider a sequence as being removed if the on-target R3 frequency is reduced after counterselection. We find that molecular counterselection is less efficient at removing off-target binders than computational counterselection and that it also removes certain on-target binders that are specific. For example, trastuzumab molecular counterselection exhibits a large false negative rate for nonspecific binders for antibody sequences that are positively enriched in cross-panning (nonspecific binders) and a large false positive rate when antibody sequences are negatively enriched in cross-panning (specific binders). In comparison, computational counterselection removed almost no specific binders and substantially removed nonspecific binders (Figure 2B).

Finally, we experimentally confirmed model predictions of nonspecific binding with a total of 48 selected on- and off-target candidates with Octet or ELISA individual binding assays. For both directions (omalizumab or trastuzumab as the off target), we grouped antibody sequences by their single-target R3 frequency into three groups: strong (top binders), medium, and weak binders. Within each group, we selected eight sequences that were not in the training set of the computational model. Where there were more than eight sequences that satisified these conditions, a random subset of eight was chosen. This resulted in a total of 48 sequences to test. We used the Octet assay for anti-trastuzumab antibodies and ELISA for anti-omalizumab, as Octet failed to produce good-quality data for the latter (STAR Methods). We observed that computational counterselection more accurately predicted ground-truth labels derived from Octet/ELISA assays when compared with molecular counterselection (Figure 2C). We next evaluated the 48 sequences using data from Fab binding and cross-panning experiments and found that computational counterselection produced superior results. We calculated the ratio of observed Fab/full-target binding, where a lower ratio indicates nonspecific binding. We also calculated the ratio of counterselection enrichment to on-target enrichment, where a higher metric indicates nonspecific binding. We found that the classifications provided by our computational model had a more accurate distribution of these nonspecificity metrics compared with classifications based upon molecular counterselection (Figure 2D).

### Generally polyspecific sequences share features with nonspecific binders to trastuzumab and omalizumab

While we find that computational counterselection is highly effective when high-throughput affinity data for off targets are available, it is possible that potential off targets are unknown or that off-target affinity data are not available. It has been previously hypothesized that nonspecificity can be characteristic to some sequences rather than being unique to pairs of on- and off targets (Cunningham et al., 2021; Notkins, 2004). This implies that a subset of antibody sequences that are found to be nonspecific could potentially be generally polyspecific—binding promiscuously to a wide range of targets—which is a highly undesirable characteristic for therapeutic use and usually only detected late in the discovery process. In these late stages of preclinical antibody development, panels of unrelated targets are used to eliminate antibody sequences that exhibit this general polyspecificity, and previous work has proposed a library with limited nonspecific sequences by identifying features that are shared among generally polyspecific sequences (Kelly et al., 2017).

**Figure 3:**
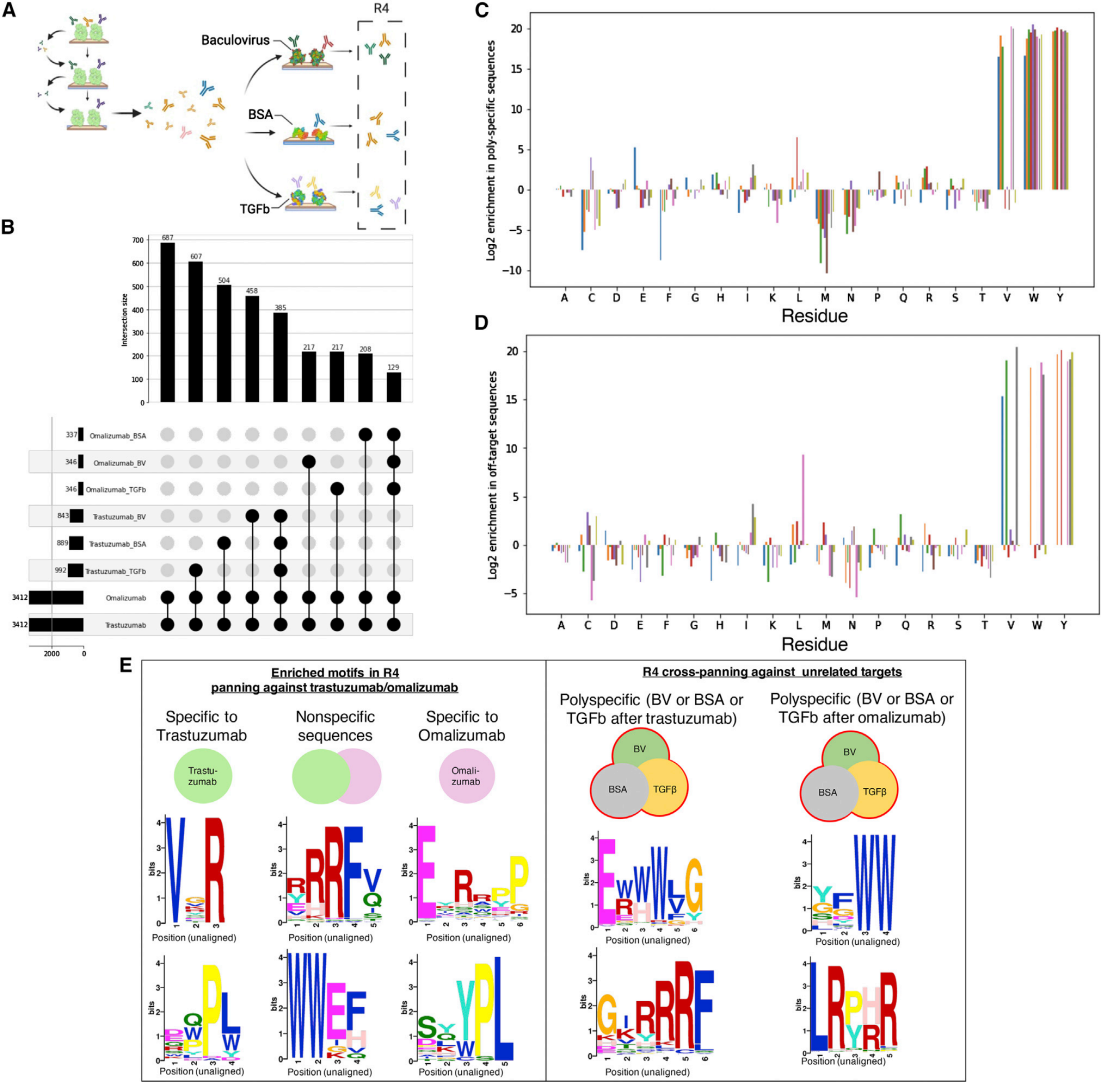
Off-target binders can be identified via cross-panning with unrelated targets (A and B) Overview of cross-panning experiments against BV extract and BSA. (B) Overlap of specific sequences identified by R4 on target and cross-panning against unrelated targets. Black circles connected by lines indicate a set made up of sequences denoted by left text labels, and bars reflect the number of members in that set. (C) Amino acid enrichment over 10 internal CDR-H3 positions (colored bars) for sequences identified to be polyspecific via panning against unrelated targets. (D) Amino acid enrichment over 10 internal CDR-H3 positions (colored bars) for sequences identified to be nonspecific via cross-panning experiments with Herceptin/Xolair. (E) Top two enriched motifs of sequences specific to trastuzumab/omalizumab, nonspecific sequences identified by cross-panning against trastuzumab/omalizumab, and polyspecific sequences identified by panning against BSA, BV extract, and TGF-β.

We hypothesized that we could train a general model for computational counterselection using affinity data from randomly selected, unrelated targets with potential affinity for a wide range of sequences as a consequence of biophysical properties, macromolecular composition, or function in biological experiments. To test this hypothesis, we conducted cross-panning (STAR Methods) against a set of unrelated targets using the R3 output of the previously described panning against trastuzumab and omalizumab. We choose three targets, baculovirus (BV) extract, bovine serum albumin (BSA), and transforming growth factor β (TGF-β). BV extract is a mix of proteins, DNA, and lipids that has been previously used for identifying polyspecific sequences, TGF-β is an extremely hydrophobic protein that adheres strongly to surfaces, and BSA is routinely used as a blocking agent (e.g., in ELISA). An additional round of panning against omalizumab and trastuzumab was also conducted (Figure 3A). We define “nonspecific” as sequences that were identified as binding both omalizumab and trastuzumab in R4 and “polyspecific” as sequences that bind any of the three unrelated targets in R4 (STAR Methods). We then found that the overlap between sequences identified as nonspecific and polyspecific sequences is large (Figure 3B). We next characterized sequences that were identified as nonspecific versus polyspecific by computing amino acid enrichment analyses and STREME motif enrichment analysis from the DREME suite of bioinformatics tools (Bailey, 2021). Additional details on this analysis can be found in the STAR Methods. Nonspecific and polyspecific sequences share similar amino acid compositions in the internal 10 positions of the CDR-H3 sequence (Figures 3C and 3D). Further, the top 2 motifs (excluding canonical antibody CDR-H3 signatures) identified by STREME are similar between polyspecific and nonspecific sequences and reflect common 2-mer motifs enriched in nonspecific sequences, as previously identified (Kelly et al., 2017) (Figure 3E). These pieces of evidence suggest that using unrelated targets along with computational counterselection could be viable for identifying generally polyspecific sequences in the absence of single-target panning data against targets of interest.

### Computational counterselection with unrelated targets identifies nonspecific binders

**Figure 4:**
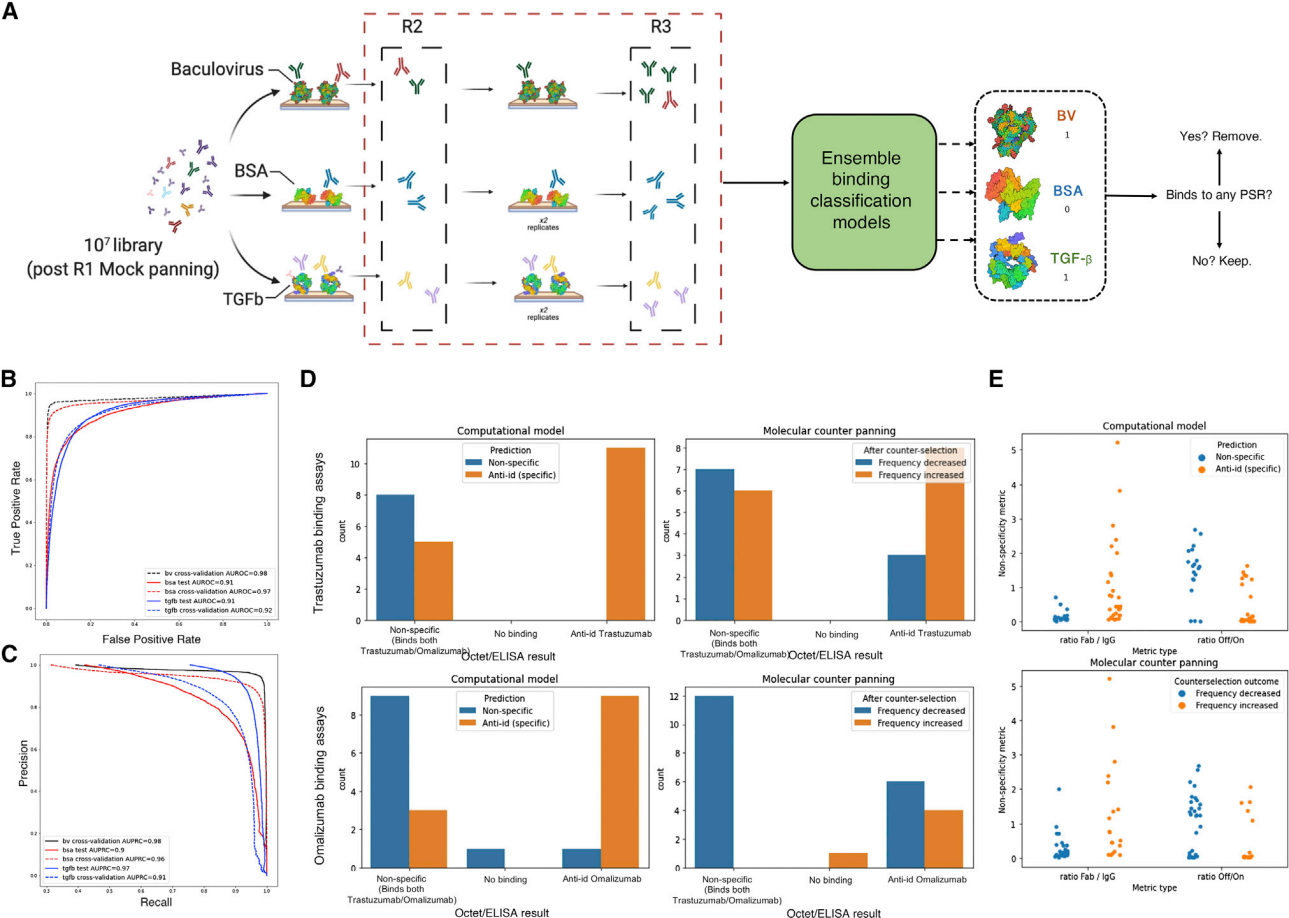
Computational counterselection models trained on unrelated targets can identify nonspecific sequences (A) Overview of single-target panning experiments against BV extract, BSA, and TGF-β. (B) AUROC curves for classifiers trained on BV extract (black), BSA (red), and TGF-β (blue). Solid lines indicate the test set is a held-out biological replicate. Dashed lines show the result of cross-validation. (C) Precision-recall curves for ensemble classifier trained on BV extract (black), BSA (blue), and TGF-β (red). Solid lines indicate the test set is a held-out biological replicate. Dashed lines show the result of cross-validation. (D) Computational counterselection with unrelated targets provides superior classifications using ground-truth metrics from Octet/ELISA data for 48 candidates split by trastuzumab (top) and omalizumab (bottom) predictions. Computational counterselection (left panel) and molecular counterselection (right panel) predictions indicated by blue (nonspecific) and orange (anti-idiopathic) bars grouped by ground-truth labels. (E) Fab/IgG (lower ratio indicates nonspecific binding) and cross-selection/on-target selection ratios (higher indicates nonspecific binding). Computational (top) and molecular (bottom) counterselection predictions indicated by blue (nonspecific) and orange (specific) were evaluated by their Fab/IgG and cross-selection/on-target selection ratio distributions.

Next, we sought to show that computational counterselection models trained on sequencing data from our three unrelated targets (BV extract, BSA, and TGF-β) are able to identify nonspecific binders. We conducted two rounds of affinity selection against BV, BSA, and TGF-β using the output of a round of panning against no target (mock) (Figure 4A). At each round, enriched antibodies were sequenced, and round enrichment was computed and given a binary labeled based on R2 to R3 enrichment. Using these data, we then trained individual binding classification models using the same architecture as described in Table S1 to predict binder or non-binder labels for each unrelated target and show that these models successfully classify binders by computing the area under the receiver operating curve (AUROC) and area under the precision-recall curve (AUPRC) for both 10-fold cross-validation on the training set and testing on a held-out biological replicate (Figures 4B and 4C).

We then performed computational counterselection using these ensemble models trained on affinity data to the three general unrelated targets (BV, BSA, and TGF-β). We labeled a sequence as nonspecific if it was predicted to bind to any one of these three unrelated targets (Figure 4A). To validate the generalizability of this computational counterselection method with unrelated targets, we repeated the experiments identifying nonspecific binders using cross-panning data and ELISA/Octet data against omalizumab and trastuzumab (and vice versa). Computational counterselection with unrelated targets outperformed molecular counterselection on the ELISA/Octet ground-truth-label prediction task (Figure 4D). Further, we found that the classifications provided by our computational model had a more accurate distribution of the Fab/full-target binding ratio and the ratio of counterselection enrichment to on-target enrichment (as previously described) compared with classifications based on molecular counterselection (Figure 4E).

## Discussion

Biologics must have both high affinity and specificity for their desired targets to be effective and safe. Sequence-based therapeutics, such as monoclonal antibodies and oligonucleotide aptamers, are often discovered via affinity-selection experiments to identify a pool of lead candidates. Screening out nonspecific binders typically occurs late in the therapeutic-development pipeline, potentially resulting in wasted time and resources on ultimately nonspecific, and therefore undevelopable, sequence candidates. Techniques such as molecular counterselection can be used during affinity-selection experiments but are not definitive and, more importantly, are experimentally costly due to the combinatorial nature of screening for all possible off targets. In this work, we introduce computational counterselection to identify nonspecific sequences without the need for combinatorial experiments.

Computational counterselection utilizes high-throughput sequencing data from affinity-selection experiments against individual targets to filter nonspecific sequences from a pool of candidates via machine learning models of affinity. Here, we conducted computational counterselection for antibody discovery and compared performance with corresponding molecular counterselection experiments. Using neural networks that predict antibody binding affinity based on phage panning enrichment over rounds of selection, we show that it is possible to filter nonspecific sequences for specific targets. In addition, we show that computational counterselection models trained on randomly selected, unrelated targets can identify nonspecific sequences without the need for off-target affinity data. Thus, computational counterselection is a tool that can be used to efficiently identify nonspecific sequences using historical and universal affinity data in place of combinatorial molecular competitor screens. The utility of computational counterselection will continue to increase as data accumulate from successive antibody discovery campaigns, providing one method of identifying highly specific antibody sequences. Further, computational counterselection can be used for any sequence-based therapeutic discovery from T cell receptors (TCRs), viral tropism targeting by capsid sequence selection, to oligonucleotide therapeutics.

### Limitations of the study

The primary limitation of present work is the need for sequence enrichment data for on- and off targets. We note that antibody discovery campaigns increasingly generate sequencing data for downstream use in analysis. We present a general strategy for identifying general polyspecificity by the reuse of these data across antibody discovery campaigns. Another limitation was the validation of our approach using commercial antibodies as targets. While their clear binding epitopes are advantageous for outlining the validity of computational counterselection, we did not test on therapeutic targets that might have multiple binding epitopes with a wider range of affinities. However, the advantage of our ensemble machine learning models is that one can directly compute uncertainty in predictions, which can be considered when evaluating thresholds for filtering nonspecific candidates. Further, we show the performance of machine learning models of affinity on large, multi-epitope antigen targets when predicting general polyspecificity. We also choose to focus on the CDR-H3 sequence because the diversified library that our data were based on focused on these positions of the antibody. Finally, an aspect of antibody therapeutic design that is not incorporated in this work is downstream affinity maturation and engineering that can alter the specificity profile of screened candidates (Mason et al., 2021). We view computational counterselection as a tool to improve the specificity distribution of candidates that advance to the lower throughput affinity maturation and engineering stage of development.

## STAR★Methods

### Key resources table

**Table:** 

REAGENT or RESOURCE	SOURCE	IDENTIFIER
**Antibodies**		

Trastuzumab	pharmacy	Herceptin®
Omalizumab	pharmacy	Xolair®

**Bacterial and virus strains**		

Helperphage VCSM13	Agilent	200251
*E. coli* TG1 F+ supE thi-1 Δ(lac-proAB) Δ(mcrB-hsdSM)5(rK– mK–) [F′ traD36 proAB lacIqZΔM15]	Roche	1 411 446 001

**Chemicals, peptides, and recombinant proteins**		

TGFb-1	Peprotech	100-21
Bovine serum albumin	Biowest	P6154
Superblock®	Thermo Scientific	37515
10 mM Glycine pH 2.0	GE Healthcare	BR-1003-55
FlashGel DNA Marker 100-4000 bp	Lonza	50473
FlashGel Loading Dye	Lonza	50462
SyBR Safe	Invitrogen	S33112
6X loading dye	Fermentas	R0611
O’GeneRuler 100 bp DNA ladder plus	Thermo Scientific	SM1153
Agarose	Invitrogen	16500-500
ChromaLink Biotin (DMF Soluble)	Solulink	B-1001-010
Neutravidin	Thermo	3100
AttoPhos™ fluorescence substrate	Roche	11 681 982 001
StreptAvidin-AP conjugated	Roche	11089 161 001
10x Kinetic buffer	ForteBio	18-1105

**Critical commercial assays**		

Qiaprep Spin Miniprep Kit	Qiagen	27106
1.2% FlashGel cassette	Lonza	57023
MiSeq® v3 Reagent Kit 150 Cycles PE	Illumina	Box1: 15043893 Box2: 15043894
Zeba Spin Desalting Columns, 2mL	Thermo	89889
Anti-Streptavidin biosensors	ForteBio	18-5019
KAPA HiFi HotStart PCR kit	Roche	07958935001
Baculovirus particle (BVP) production for polyspecificity screening kit	Lake Pharma	25690
Wizard® SV Gel and PCR Clean-Up System	Promega	A9282

**Deposited data**		

Training data for computational counterselection	This paper	doi:10.5281/zenodo.6625509

**Oligonucleotides**		

TruSeq_for_fused PCR: NL15 AATGATACGGCGACCACCGAGATCTACAC TCTTTCCCTACACGACGCTCTTCCGATCTt gtattattgcgcgcgt	Microsynth	N/A
TruSeq_for_fused PCR: NL22 AATGATACGGCGACCACCGAGATCTACAC TCTTTCCCTACACGACGCTCTTCCGATCT Atgtattattgcgcgcgt	Microsynth	N/A
TruSeq_for_fused PCR: NL23 AATGATACGGCGACCACCGAGATCTAC ACTCTTTCCCTACACGACGCTCTTCCG ATCTCAtgtattattgcgcgcgt	Microsynth	N/A
TruSeq_for_fused PCR: NL24 AATGATACGGCGACCACCGAGATCTA CACTCTTTCCCTACACGACGCTCTTCC GATCTGAAtgtattattgcgcgcgt	Microsynth	N/A
TruSeq_index5_rev_fused PCR: NL35 CAAGCAGAAGACGGCATACGAGATCA CTGTGTGACTGGAGTTCAGACGTGTG CTCTTCCGATCTtgaccacgctgctcagg	Microsynth	N/A
TruSeq_index6_rev_fused PCR: NL36 CAAGCAGAAGACGGCATACGAGATAT TGGCGTGACTGGAGTTCAGACGTGT GCTCTTCCGATCTtgaccacgctgctcagg	Microsynth	N/A
TruSeq_index12_rev_fused PCR: NL42 CAAGCAGAAGACGGCATACGAGATTA CAAGGTGACTGGAGTTCAGACGTGTG CTCTTCCGATCTtgaccacgctgctcagg	Microsynth	N/A
TruSeq_index19_rev_fused PCR: NL48 CAAGCAGAAGACGGCATACGAGATT TTCACGTGACTGGAGTTCAGACGTG TGCTCTTCCGATCTtgaccacgctgctcagg	Microsynth	N/A

**Recombinant DNA**		

Single framework library	Liu et al. 2020	doi:10.1093/bioinformatics/btz895

**Software and algorithms**		

Computational counterselection	This paper	doi:10.5281/zenodo.6625278
Gen5 3.08	Biotek software	https://www.biotek.com/products/software-robotics-software/gen5-microplate-reader-and-imager-software/
GraphPad Prism 9	GraphPad	https://www.graphpad.com/updates/prism-900-release-notes
Python v1.9.1	Python	python.org
PyTorch v1.7.1	Meta	pytorch.org

**Other**		

Qubit	Invitrogen	Q32866
MiSeq system	Illumina	N/A
ForteBio Data Analysis 9.0	ForteBio	N/A

### Resource availability

#### Lead contact

Further information and requests should be directed to and will be fulfilled by the lead contact, David K. Gifford (gifford@mit.edu).

#### Materials availability

This study did not generate new unique reagents.

### Method details

#### Identification of specific and nonspecific antibodies via panning experiments

We performed single-target phage-panning, cross-panning, and molecular counterselection experiments. In the single-target experiments three rounds of phage panning were performed against a single target. In the cross-panning experiments, two rounds of phage-panning were performed against a first target, and the final third round of panning was performed against a second-target. Thus cross-panning selects antibody sequences that bind to both targets. In Fab-panning experiments, two rounds of panning were done against a target, and for the final third round of panning only the target’s Fab region was used. In the molecular counterselection experiments, two rounds of panning were done against a target, and the final third round was performed in the presence of a competitor molecule that inhibited isolation and sequencing of antibodies bound to the competitor.

In all phage-panning experiments a single framework, randomized library was used. The library used in all selection experiments is the same as was used in Liu et al., 2020 (Liu et al., 2020). A gene fragment encoding the germline framework combination IGHV3-23 and IGKV1-39 was synthesized by Invitrogen’s GeneArt service in Fab format and cloned into a phagemid vector serving as the base template. IGHV3-23 and IGKV1-39 were used as they display a favorable framework combination for a phage display library and this framework combination is used in several therapeutic antibodies including trastuzumab and bevacizumab (Tiller et al., 2013). The phagemid vector consists of Ampicillin resistance, ColE1 origin, M13 origin and a bi-cistronic expression cassette under a lac promotor with OmpA - light chain followed by PhoA–heavy chain – Amber stop – truncated pIII (amino acids 231 – 406). Only CDR-H3 was diversified and primers were designed to incorporate all naturally occurring amino acids excluding cysteine (free cysteines could form disulfide bonds), and asparagine (asparagine in conjunction with certain amino acids could undergo deamidation or become glycosylated) using trinucleotide technology (ELLA Biotech). CDR-H3 lengths between 10 and 16 amino acids and 18 amino acids were allowed, in which the last two amino acids were kept constant with the sequence Asp-Tyr for length 10 to 16 and Asp-Val for length 18. The design of the final two CDR-H3 amino acids reflects human VDJ recombination. Short CDR-H3s more often use J- fragment IGHJ4 with “DY” at the end of CDR-H3 while longer CDR-H3s (here 18 aa) more often use IGHJ6 with “DV” at the end of CDR-H3. Library inserts were generated by PCR using Phusion High Fidelity DNA polymerase (NEB Biolabs). The resulting CDR-H3 library inserts were ligated into the base template, transformed into E.coli TG1 DUO (Lucigen) with a minimal library size of 1E+09 transformants per CDR-H3 length and phages were produced using M13KO7 helper phage (NEB Biolabs) using standard previously described protocols (Proetzel and Ebersbach, 2016).

Panning of the library against the targets (in-house expression and purification) was done in solid phase mode. 96-well maxisorb plates (Nunc) were coated with the target using 500 nM in first and second round and 200 nM in the third round. After each round of phage selection, polyclonal plasmid DNA was prepared using QIAprep Spin Miniprep Kit (Qiagen). Samples were analyzed on a MiSeq using MiSeq Reagent Kit v3 (Illumina) with 150 forward cycles or on a HiSeq using HiSeq PE Cluster Kit v4 cBot and HiSeq SBS Kit v4 (Illumina) with 76 forward cycles. For all targets, a replicate experiment was performed.

#### Description of panning targets

For initial on-target and off-target counterselection validation, we choose two publicly available antibody targets, trastuzumab (monoclonal antibody that binds to human epidermal growth factor receptor 2) (Hudis, 2007) and omalizumab (monoclonal antibody that binds to IgE antibodies) (Davies et al., 2017) because they have both shared and unique binding epitopes that provide an interpretable source of nonspecificity. We chose commercial antibodies because they provide a clear mechanism for both specific and nonspecific binding, and when introducing our method and comparing to molecular counterselection we wanted to clearly show that the experimental readout observed in our validation datasets (cross-panning and low-throughput ELISA/Octet) are indicative of specificity and not other sources of noise (i.e spectrum of sequence similarity of binding epitopes between commercial antigens). Because the epitope of each antibody is guaranteed to be highly specific when compared with the other, antibody binding to the variable region is either driven by highly specific interaction or general polyspecificity (which motivates latter sections of the paper). Similarly, nonspecific binding can be assumed to come from binding to the Fc region of the antibody or general polyspecificity. This ensures that the advantage of computational counterselection over molecular counterselection on validation tasks is not due to spurious signals that are picked up from the model and reflected in the validation datasets for trastuzumab/omalizumab non-specific binding. We note that we use unrelated targets that are commercially available sticky antigens to address the source of nonspecificity potentially being polyspecific sequences (another signal that using antibodies as targets does not mitigate well). We then chose 3 unrelated antigen targets to train a general polyspecificity predictor (described in the main text).

#### High throughput sequencing data processing and training dataset information

For high throughput sequencing of antibodies form all selection experiments, in each experiment we had around 10^7^ high quality (Figure S3) sequences from all 3 rounds of panning. To extract CDR-H3 regions, the fixed flanking sequence of the variable regions (12 base pairs on the head and 9 base pairs on the tail) were used as a template to locate and segment out the CDR-H3 sequence. BLAST (Altschul et al., 1990) was used for short read alignment to align the template with each read, allowing a maximum of 3 mismatches on each side. We then took the sequence between the end of the head and tail template and extracted sequences that were multiples of three, indicating translated codons. The translation to amino acid sequences was done using EMBOSS (Rice et al., 2000).

Training datasets were constructed by retaining sequences that had more than 3 read counts in at least one panning round, or had non-zero reads in all rounds. For trastuzumab/omalizumab multi-task regression datasets, R2-to-R3 enrichment was used as the label. Sequences from each target affinity selection experiment were processed independently and labels were then concatenated, allowing missing values across targets. This resulted in a dataset of 68,943 sequences. ∼7,000 sequences of this dataset were held-out for testing. For the unrelated targets individual classification datasets, sequences with a round 3 frequency greater than 5e-5 or that is higher than its round 2 frequency by more than 1e-6 was labeled as positive, while sequence whose round 3 frequency is lower than round 2 frequency by more than 1e-6 and whose round 3 frequency is less than 5e-5 was labeled as negative. This resulted in a training dataset of 40,129 sequences for TGFβ with 21,430 non-binders and 18,699 binders, 25,669 sequences for BSA with 17,629 non-binders and 8,040 binders, and 30,998 sequences for Baculovirus with 19,011 non-binders and 11,987 binders. Using a biological replicate, test sets for BSA and TGFβ were labelled the same way resulting in test sets of 25,669 (8,909 non-binders/6,329 binders) and 40,129 (6,214 non-binders/19,205 binders) sequences, respectively. Baculovirus did not have a biological replicate and models were evaluated using 10-fold cross-validation.

#### Training neural network ensembles for predicting binding affinity

Our machine learning models input the complementarity-determining region heavy-chain three (CDR-H3) sequence of a Fab molecule and output the predicted binding of the Fab to a target or a binary classification label of “binder” or “non-binder”. We train each model on high-throughput data from subsequent rounds of one or more phage panning experiments against the target as previously described.

We used six different deep learning architectures for our network ensemble models of antibody binding for both regression and classification tasks (ensembled by average or voting, respectively). Five were convolutional neural networks with 1 or 2 convolutional layers with filter size of 1, 3 or 5 residues and stride 1, followed by a local max-pooling layer with window size 2 and stride 2. We used 64 and 32 convolutional filters for single convolutional layer networks. In one of the double convolutional layer networks, we used 32 filters with width 5 in the first layer and 64 filters with width 5 in the second layer. In the other network, we used 8 convolutional filters with width 1 in the first layer to learn an embedding from one-hot to hidden space for each amino acid, and then used 64 filters with width 5 to learn higher level patterns. In each of our convolutional models, the output from the last convolutional layer was fed into a fully connected layer with 16 hidden units and a dropout layer. It is then connected to the final output layer that outputs predictions for each of the target antigens. Our sixth architecture was a 2-layer fully connected neural network with 32 hidden units and dropout in each layer. Table S1 the detailed setup of each architecture and the number of parameters in each architecture. Each model was trained using Adam optimizer with default PyTorch v1.7 parameters (Paszke et al., 2019). Model performances were evaluated using the validation set after each epoch, and the model with the highest performance was saved. All models were trained using either a single NVIDIA Titan RTX GPU (24 GB RAM) or a single GeForce GTX 1080 Ti (11 GB RAM).

#### Individual binding assays for specificity validation

Biotinlyation of trastuzumab and omalizumab and all predicted anti-omalizumab binders was performed using ChromaLink Biotin (DMF Soluble) from Solulink according to supplier’s manual. ChromaLink biotin stock solution was prepared using DMSO. Each reaction was performed with 10 equivalent of biotin (90 min at RT). Biotinylated proteins were dialysed using Zeba Desalting column 2 mL following supplier’s protocol. PBS was selected as final buffer.

To confirm binding prediction to trastuzumab and Fc, Octet® was performed using biotinlyated targets loaded on StreptAvidin biosensors. StreptAvidin biosensors were first equilibrated in 1X kinetics buffer (90 μL per well) in a 384 black wells plate during 5 min. Biosensors were then dipped into biotinylated targets (trastuzumab, omalizumab and BSA as a negative control; 100 nM in 1X kinetics buffer, 90 μL per well) during 10 min. The baseline was reached by dipping pins in 1X kinetics buffer (90 μL per well) during 5 min. Predicted anti-trastuzumab binders (12 binders) and anti-Fc binders (20 binders) at 100 nM and four additional anti-Fc binders from the predicted weak group at 400 nM (in 1X kinetics buffer, 90 μL per well) were associated to targets during 10 min and dissociated by finally dipping pins in 1X kinetics buffer (90 μL per well) for 10 min. Assay temperature was set to 25°C. Within assay, biosensors were regenerated using 3 cycles of Glycine 2.0 for regeneration steps and 1x kinetic buffer for neutralization steps. Analysis was performed with Octet data Analysis software (ForteBio Data Analysis 9.0). Responses with values above 0.10 nm were defined as binding signals. Binders are considered as anti-Fc binders when response to more than two targets was above this value.

Octet for anti-omalizumab failed because we did not see any binding for antibody sequences expected to have high affinity for omalizumab (based on panning) in contrast to high affinity anti-trastuzumab and anti-Fc sequences which worked as expected in the anti-trastuzumab Octet experiment. As described above, we biotinylated the targets and bound them to streptavidin pins. As we did random biotinylation, we did not have control of where the biotin is going. Our hypothesis is that random biotinylation of omalizumab caused predominant addition of biotin at or close by the CDRs of omalizumab and thus altering or destroying the binding epitope of omalizumab-specific candidates. In ELISA, we used the Fab version of omalizumab and coated it directly to maxisorb multi-well plates in order to maintain the binding epitope. ELISA was performed using black MaxiSorp™ 384-wells plates coated ON at 4°C with Fab-format of the targets (ranibizumab-Fab, trastuzumab-Fab, omalizumab-Fab and BSA ) at a concentration of 200 nM in PBS (20 uL per well). All following steps were performed at room temperature. After washing 2x with TBST, wells are blocked for 2 h with Superblock® (80 uL per well). Plates were washed 2x with TBST and biotinylated binders at 40 nM were added (20ul per well). Binding was allowed for 2 h. Plates were washed 3x with TBST and StreptAvidin-AP conjugated antibody (1:5000 dilution in PBST) is added (20ul per well). After 1 h incubation, plates were washed 5x with TBST and 20ul per well of AttoPhos® substrate 1/5 diluted in water were added. Plates were read after 5 min incubation in the dark using an excitation wavelength of 430 nm and an emission wavelength of 535 nm using BioTek Synergy neo2 (multi-mode reader) with Gen5 3.08 software. Binding was defined when signal at 40 nM was at least 5 times over background signals.

#### Details of motif enrichment analysis on post-trastuzumab/omalizumab output

Panning was done for a fourth round after three rounds of panning against trastuzumab and omalizumab, respectively. Panning for a fourth round was conducted following the same procedure described above and data processing was conducted as described above. To reduce noise, only sequences with at least 1 read count in a round of panning were retained. The set of specific sequences to trastuzumab or omalizumab were identified with stringent filtering of having a round 3 to round 4 fold-change of greater than 2.0. “Nonspecific” or “off-target” sequences were the overlap of these lists of trastuzumab/omalizumab specific sequences with affinity for trastuzumab and omalizumab. Polyspecific sequences were identified with stringent filtering of having a round 3 to round 4 fold-change of greater than 2.0 in one of the unrelated targets, BV, BSA, or TGFβ. For all round 4 analysis, replicates were combined. Motif enrichment analysis was done using STREME with the following configuration: --patience 20 --minw 3 --maxw 6.

### Quantification and statistical analyses

All analyses were done using Python version 3.9. Machine learning model training was done using PyTorch version 1.7. Pearson r values and were computed with *scipy* (Virtanen et al., 2020). Receiver Operating Characteristic (ROC) and precision-recall (PR) curves and corresponding area under ROC/PR curves (AUROC/PR) were computed using *scikit-learn* (Pedregosa et al., 2011).

## Data Availability

• Training data have been deposited at Zenodo and are publicly available as of the date of publication. DOI (Zenodo: doi:10.5281/zenodo.6625509) is listed in the key resources table. • Code has been deposited at Github and Zenodo and is publicly available as of the date of publication. DOI (Zenodo: doi:10.5281/zenodo.6625278) is listed in the key resources table. • Any additional data required to reanalyze the data reported in this work paper is available from the lead contact upon request.

## Supplemental information

Document S1. Table S1 and Figures S1–S3

Document S2. Article plus supplemental information
