# Supplementary material for: Computational counterselection identifies nonspecific therapeutic biologic candidates
Source: Cell Rep Methods. 2022 Jul 11;2(7):100254. doi: 10.1016/j.crmeth.2022.100254 (PMC9308162; doi:10.1016/j.crmeth.2022.100254)
Supplement: Document S1. Table S1 and Figures S1–S3 [file mmc1.pdf]

Cell Reports Methods, Volume *2*

Supplemental information

Computational counterselection identifies nonspecific therapeutic biologic candidates

Sachit Dinesh Saksena, Ge Liu, Christine Banholzer, Geraldine Horny, Stefan Ewert, and David K. Gifford

Supplemental Information

Computational counterselection identifies nonspecific therapeutic biologic candidates

Sachit D. Saksena, Ge Liu, Christine Banholzer, Geraldine Horny,
Stefan Ewert, David K. Gifford

	Num. of convolutional layers	Conv 1	Conv 2	Num. of fully connected layer	Num. of Fully connected neurons
Seq_32_32	0	N\A	N\A	2	32
Seq_32x1_16	1	Width 5, 32	N\A	1	16
Seq_32x2_16	2	Width 5, 32	Width 5, 64	1	16
Seq_64x1_16	1	Width 5, 64	N\A	1	16
Seq_32x1_16_filt3	1	Width 3, 32	N\A	1	16
Seq_embed_32x1_16	2	Width 1, 8	Width 5, 32	1	16

Table S1: Ensemble binding affinity machine learning model details. Related to Figures 1, 2, and 4 in the main text.

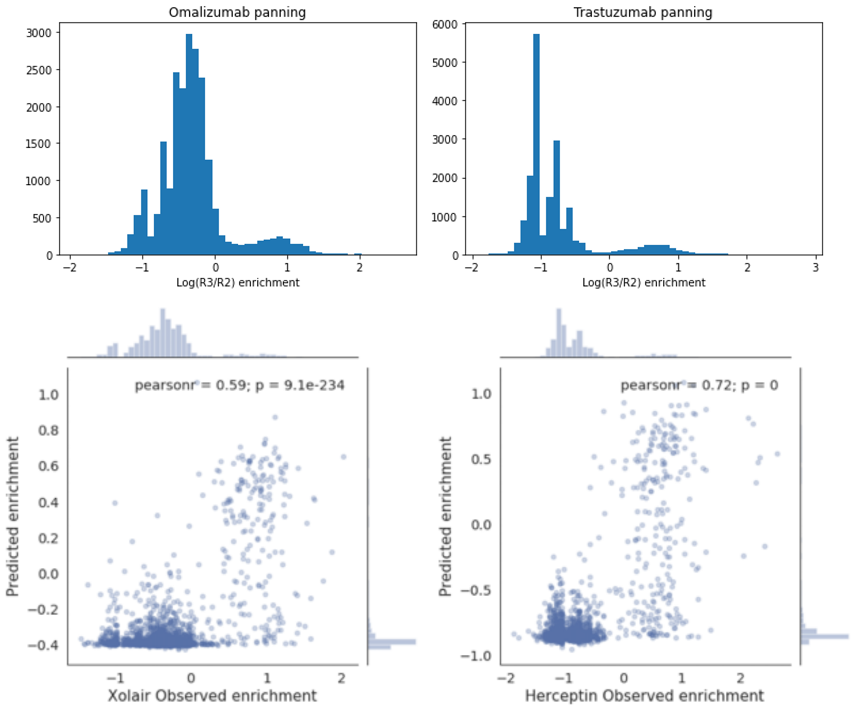

Figure S1: Top: Round-enrichment distribution of regression labels for trastuzumab and omalizumab training data. Bottom: Ensemble multi-task affinity models prediction performance on held-out data for omalizumab (left) and trastuzumab (right). Related to models and data used in Figure 2.

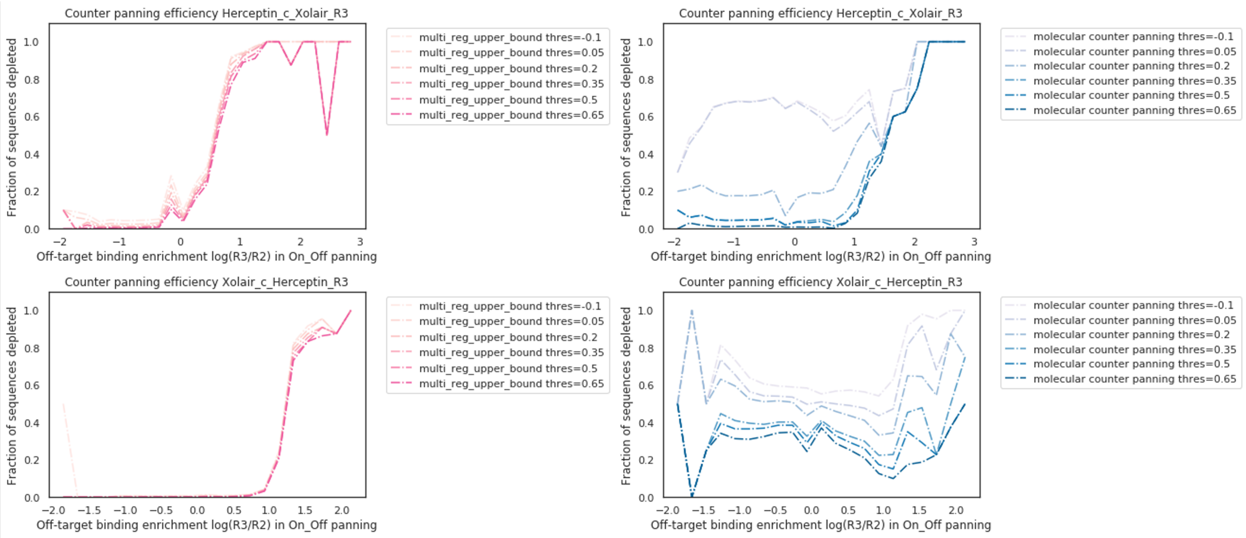

Figure S2: Selecting thresholds for computational counterselection (left) and molecular counterselection (right) on crosspanning data for trastuzumab and omalizumab. Computational counterselection outperforms molecular counterselection across a range of thresholds. Related to Figure 2.

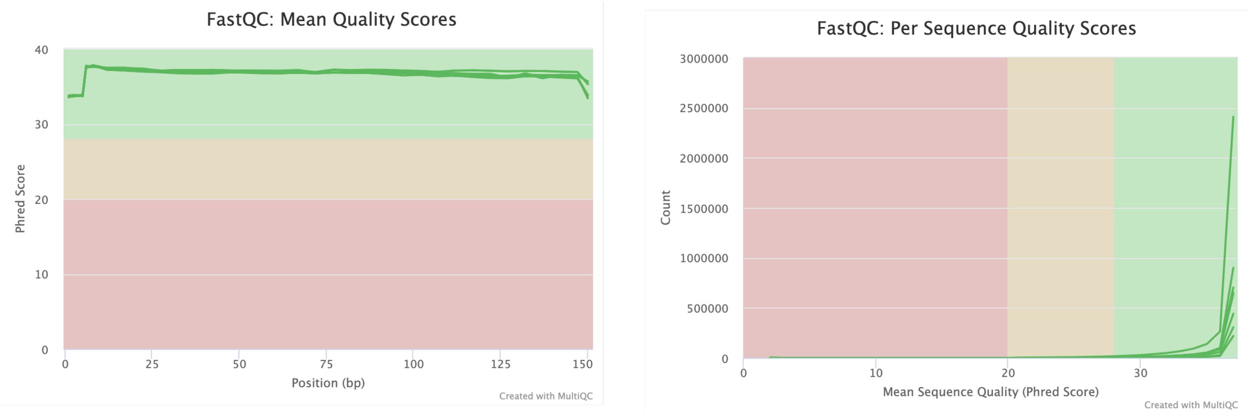

Figure S3: Sequencing quality for all training datasets (multiple lines). Per base sequence quality showing the average quality across each base position across reads in each sample (left). Per sequence quality scores showing the number of reads with average quality scores. Related to Figure 2, 3, and 4.
